# Sequencing and association analysis of the type 1 diabetes – linked region on chromosome 10p12-q11

**DOI:** 10.1186/1471-2156-8-24

**Published:** 2007-05-17

**Authors:** Sergey Nejentsev, Luc J Smink, Deborah Smyth, Rebecca Bailey, Christopher E Lowe, Felicity Payne, Jennifer Masters, Lisa Godfrey, Alex Lam, Oliver Burren, Helen Stevens, Sarah Nutland, Neil M Walker, Anne Smith, Rebecca Twells, Bryan J Barratt, Charmain Wright, Lisa French, Yuan Chen, Panagiotis Deloukas, Jane Rogers, Ian Dunham, John A Todd

**Affiliations:** 1 Juvenile Diabetes Research Foundation/Wellcome Trust Diabetes and Inflammation Laboratory, Cambridge Institute for Medical Research, University of Cambridge, Cambridge, UK; 2 The Wellcome Trust Sanger Institute, Wellcome Trust Genome Campus, Hinxton, Cambridge, UK

## Abstract

**Background:**

In an effort to locate susceptibility genes for type 1 diabetes (T1D) several genome-wide linkage scans have been undertaken. A chromosomal region designated *IDDM10 *retained genome-wide significance in a combined analysis of the main linkage scans. Here, we studied sequence polymorphisms in 23 Mb on chromosome 10p12-q11, including the putative *IDDM10 *region, to identify genes associated with T1D.

**Results:**

Initially, we resequenced the functional candidate genes, *CREM *and *SDF1*, located in this region, genotyped 13 tag single nucleotide polymorphisms (SNPs) and found no association with T1D. We then undertook analysis of the whole 23 Mb region. We constructed and sequenced a contig tile path from two bacterial artificial clone libraries. By comparison with a clone library from an unrelated person used in the Human Genome Project, we identified 12,058 SNPs. We genotyped 303 SNPs and 25 polymorphic microsatellite markers in 765 multiplex T1D families and followed up 22 associated polymorphisms in up to 2,857 families. We found nominal evidence of association in six loci (*P *= 0.05 – 0.0026), located near the PAPD1 gene. Therefore, we resequenced 38.8 kb in this region, found 147 SNPs and genotyped 84 of them in the T1D families. We also tested 13 polymorphisms in the PAPD1 gene and in five other loci in 1,612 T1D patients and 1,828 controls from the UK. Overall, only the D10S193 microsatellite marker located 28 kb downstream of *PAPD1 *showed nominal evidence of association in both T1D families and in the case-control sample (*P *= 0.037 and 0.03, respectively).

**Conclusion:**

We conclude that polymorphisms in the CREM and SDF1 genes have no major effect on T1D. The weak T1D association that we detected in the association scan near the PAPD1 gene may be either false or due to a small genuine effect, and cannot explain linkage at the *IDDM10 *region.

## Background

Type 1 diabetes (T1D) is the second most common chronic disease in children. It develops as a result of a complex interaction of genetic and environmental factors leading to the immune-mediated destruction of the insulin-producing pancreatic β-cells. Genetic predisposition has a significant role in T1D as suggested by familial clustering of the disease and increased concordance among monozygotic twins [1]. The identification and localization of susceptibility genes for complex traits or common diseases has made slow progress, owing to many factors including small effect sizes, incomplete knowledge of the polymorphism content of the genome and its patterns of linkage disequilibrium and lack of inexpensive genotyping technologies. One approach to narrowing down to specific genome regions that might contain a susceptibility gene or genes has been to carry out linkage studies in affected sib-pair families. In contrast to monogenic diseases, this approach has had limited success in multifactorial diseases.

Nevertheless, in T1D, combined analyses of several studies provided evidence for four linked regions, the major locus MHC on 6p21 (previously designated *IDDM1*), 10p14-q11 (*IDDM10*), 2q31-q33 (*IDDM7 *and *IDDM12*) and 16q22-q24 [2]. Here we undertook analysis of sequence polymorphisms in the putative *IDDM10 *region, comprising 23 Mb region on the chromosome 10p12-q11. We have identified a large number of single nucleotide polymorphisms (SNPs) and performed an association scan of this region in a large collection of T1D families, as well as unrelated patients and controls.

## Results

Initially, in order to identify T1D genes in the *IDDM10 *region we adopted a candidate gene approach. Previously we examined the GAD2 gene, which encodes a major T1D autoantigen GAD65 protein, and found no evidence of association [3]. Here we resequenced and studied association of two candidate genes, *CREM *and *SDF1*, which also map to this region. The cyclic adenosine 5'-monophosphate responsive element modulator (CREM) has been shown to bind to the Interleukin-2 gene promoter and suppress expression of this cytokine [4], which is critical for the initiation and termination of the immune response as well as for T cell development. Increased CREM expression was found in T cells of patients with another autoimmune disease, systemic lupus erythematosus [5]. By resequencing the CREM gene we identified 32 SNPs, including 13 novel SNPs (Supplementary Table 1, see Additional file 1). We selected six tag SNPs and genotyped them in 1,612 T1D patients and 1,828 controls from the UK. We found a multi locus *P *= 0.98, indicating that common variants of *CREM *do not affect T1D susceptibility in a major way.

**Table 1:** Association analysis in the extended T1D family set.

*Gene *Polymorphism	Allele 1/2^c^	Affected offspring	Allele 1, Transmitted (%)	Allele 1, Non-transmitted (%)	*P*-value TDT	Allele 1, frequency ^d^
*PAPD1*						
Rs639299^a^	G/A	2029	777 (48.2)	834 (51.8)	0.16	0.69
rs1963187^a^	A/T	2383	272 (56.1)	213 (43.9)	0.0074	0.95
rs2480285^a^	G/A	2642	785 (46.3)	909 (53.7)	0.0026	0.21
D10S1426^a^		1401			0.21	
	*158		502 (52.7)	450 (47.3)	0.092	0.27
	*162		502 (48.4)	535 (51.6)	0.31	0.33
	*166		420 (52.3)	383 (47.7)	0.19	0.23
D10S193^a^		1932			0.037	
	*220		587 (46.6)	673 (53.4)	0.015	0.28
	*224		781 (52.2)	716 (47.8)	0.093	0.35
	*226		363 (55.1)	296 (44.9)	0.0091	0.11
	*228		185 (47.8)	202 (52.2)	0.39	0.07
rs540994 ^b^	A/C	2096	941 (51.4)	889 (48.6)	0.22	0.45
ss48399034^b^	A/T	2124	178 (58.4)	127 (41.6)	0.0035	0.96
rs1047991^b^	G/A	2382	849 (48.1)	916 (51.9)	0.11	0.73
rs11818836^b^	A/G	2087	958 (52.2)	876 (47.8)	0.056	0.45
ss48399025^b^	A/C	2284	388 (54.0)	330 (46.0)	0.030	0.90
ss48399036^b^	A/G	3207	137 (55.0)	112 (45.0)	0.11	0.02
ss48399044^b^	T/A	2061	461 (46.6)	529 (53.4)	0.031	0.83
*NRP1*						
rs13324^a^	A/G	2134	826 (50.6)	806 (49.4)	0.62	0.30
rs1048804^a^	A/G	2047	661 (47.7)	725 (52.3)	0.086	0.75
rs1010826^a^	A/G	2125	737 (51.9)	683 (48.1)	0.15	0.24
*MYO3A*						
rs1339816 ^a^	A/G	2107	820 (47.7)	898 (52.3)	0.060	0.35
rs2096176 ^a^	A/G	2116	893 (47.6)	984 (52.4)	0.036	0.42
*HNRPF*						
rs1416226^a^	A/G	2090	728 (53.5)	632 (46.5)	0.0092	0.23
*SVIL*						
rs2274494^a^	A/G	2152	894 (47.5)	987 (52.5)	0.032	0.37
rs11600^a^	A/G	2147	432 (47.5)	477 (52.5)	0.14	0.13
Intergenic						
rs787025 ^a^	A/C	2038	237 (45.6)	283 (54.4)	0.044	0.06
BMS1L						
rs787795^a^	A/G	2102	537 (53.4)	469 (46.6)	0.032	0.86

a – polymorphism genotyped in the initial scan, b – polymorphisms genotyped after being identified by resequencing of the PAPD1-MAP3K8 gene region, c – alleles encoded as 1and 2 for SNPs or as size of an allele for STRs, d – allele frequency among parents of T1D patients from the UK is shown; TDT – transmission disequilibrium test

By resequencing the cytokine stromal cell-derived factor 1 (SDF1 or CXCL12) gene we identified 33 variants, including two insertion/deletion polymorphisms, 21 of which were novel (Supplementary Table 2, see Additional file 2). We selected six tag SNPs, genotyped them in 1,612 cases and 1,828 controls and found no association (multi locus *P *= 0.67). We also tested SNP rs1801157, also known as *3'A(801G>A)*, in the evolutionary conserved 3' untranslated segment of *SDF1 *that previously had been associated with early onset of T1D [6,7]. We attempted to replicate these findings and genotyped rs1801157 in 1,800 T1D families from the UK, USA and Norway. The A allele and AA genotype frequencies were very similar to those reported previously (19.4% and 5.8%, respectively). The transmission disequilibrium test revealed no association with T1D (507 transmitted A alleles and 530 untransmitted, *P *= 0.47; relative risk for AA genotype = 1.01, 95% CI = 0.87–1.16, *P =*0.89). Even though we obtained no evidence of association we subdivided the families by age-at-onset and by *HLA-DRB1 *genotype because the two previous studies had carried out subgroup analyses. However, we found no association in any subgroup (data not shown).

**Table 2:** Association analysis in the 1,693 T1D patients and 1,805 controls from the UK.

*Gene*	Allele	Type 1 diabetes patients			Controls			MAF			
		
Polymorphism	1/2	11 (%)	12 (%)	22 (%)	11 (%)	12 (%)	22 (%)	T1D	Controls	OR for allele 1 (95% CI)	*P*-value
*PAPD1*											
rs2480285	G/A	82 (5.0)	544 (33.4)	1002 (61.5)	59 (3.4)	600 (34.9)	1060 (61.7)	0.22	0.21	1.05 (0.94 – 1.18)	0.39
ss48399025	A/C	1375 (81.4)	296 (17.5)	19 (1.1)	1469 (81.4)	326 (18.1)	10 (0.6)	0.10	0.1	0.97 (0.83 – 1.13)	0.68
ss48399034	A/T	1528 (90.6)	155 (9.2)	3 (0.2)	1620 (92.3)	133 (7.6)	2 (0.1)	0.05	0.04	0.81 (0.64 – 1.02)	0.076
ss48399036	A/G	0 (0.0)	64 (3.8)	1629 (96.2)	0 (0.0)	60 (3.3)	1734 (96.7)	0.02	0.02	1.13 (0.79 – 1.62)	0.49
rs1963187	A/T	1457 (89.1)	170 (10.4)	8 (0.5)	1564 (89.6)	175 (10.0)	7 (0.4)	0.06	0.05	0.95 (0.77 – 1.17)	0.62
D10S193	226/non 226	21 (1.3)	293 (18.5)	1270 (80.2)	15 (1.0)	261 (16.6)	1297 (82.5)	0.11	0.09	1.16 (0.98 – 1.37)	0.078
D10S193	228/non 228	4 (0.3)	158 (10.0)	1422 (89.8)	13 (0.8)	195 (12.4)	1365 (86.8)	0.05	0.07	0.73 (0.59 – 0.90)	0.0031
*NRP1*											
rs1048804	A/G	962 (57.7)	605 (36.3)	100 (6.0)	1068 (59.9)	636 (35.7)	80 (4.5)	0.24	0.22	0.9 (0.81 – 1.01)	0.071
*MYO3A*											
rs1339816	A/G	183 (11.3)	686 (42.3)	751 (46.4)	190 (11.2)	731 (43.1)	774 (45.7)	0.32	0.33	0.99 (0.89 – 1.09)	0.79
rs2096176	A/G	312 (18.7)	790 (47.2)	570 (34.1)	310 (17.5)	872 (49.1)	593 (33.4)	0.42	0.42	1.01 (0.92 – 1.11)	0.83
*HNRPF*											
rs1416226	A/G	101 (6.1)	574 (34.4)	993 (59.5)	91 (5.2)	629 (36.0)	1028 (58.8)	0.23	0.23	1.0 (0.90 – 1.12)	0.95
*SVIL*											
rs2274494	A/G	230 (13.7)	784 (46.9)	659 (39.4)	214 (12.2)	847 (48.2)	698 (39.7)	0.37	0.36	1.04 (0.94 – 1.15)	0.42
rs11600	T/C	32 (1.9)	372 (22.3)	1261 (75.7)	25 (1.4)	397 (22.3)	1362 (76.3)	0.13	0.13	1.05 (0.91 – 1.21)	0.48
Intergenic											
rs787025	T/G	8 (0.5)	226 (13.6)	1433 (86.0)	6 (0.3)	237 (13.2)	1549 (86.4)	0.07	0.07	1.05 (0.87 – 1.26)	0.62

Abbreviations: CI, confidence interval; MAF, minor allele frequency; OR, odds ratio; SNP, single nucleotide polymorphism.

We then conducted a comprehensive genetic analysis of the whole *IDDM10 *region in order to systematically identify new T1D gene(s). As part of the Human Genome Project (HGP) the Wellcome Trust Sanger Institute constructed a single tile path, i.e. set of overlapping BACs derived from two different libraries [8,9]. The overlaps between clones in this tile path were checked for SNPs and those were deposited in the dbSNP database previously [10]. In order to discover additional novel SNPs in the *IDDM10 *region we constructed a second tile path that covers the whole region using clones from both BAC libraries, so that finished genome sequence from one library was complemented by a clone from the second library, i.e. from a different individual. This second tile path was then shotgun sequenced. Thus, we revealed additional polymorphic sites located outside BAC overlaps in the initial HGP tile path. In total we identified and submitted to dbSNP 12,058 SNPs, of which 10,808 were uniquely mapped onto the human genome build 34, including 1,320 SNPs that were novel, i.e. not present in dbSNP build 120. These SNPs contributed substantially to the polymorphism content of the *IDDM10 *region.

We then screened for association with T1D sequence polymorphisms between 21.0 Mb and 44.3 Mb of chromosome 10 (NCBI genome build 34) that include the *IDDM10 *region. In total 303 SNPs and 25 polymorphic microsatellite markers/short tandem repeats (STRs) were genotyped in up to 765 families with two affected offspring (Supplementary Table 3, see Additional file 3). This sample includes families in which linkage of *IDDM10 *was characterized initially [11-13]. We found 14 polymorphisms in nine loci showing nominal evidence of association with T1D (*P *< 0.05). In order to investigate these results further, we genotyped these polymorphisms in an additional set of T1D families (Table 1). In the combined analyses of up to 2,857 families we found some evidence for T1D association of D10S193, rs1963187 and rs2480285 (*P *= 0.037, 0.0074 and 0.0026, respectively), which are clustered in a 97 kb region (coordinates: chr10; 30,577,375..30,674,697; NCBI genome build 34). Their association with the disease was largely independent of each other (between rs1963187 and rs2480285 r^2 ^= 0.24, while between risk associated allele 226 of D10S193 and rs1963187 and rs2480285 r^2 ^= 0 and 0.01, respectively).

**Figure 1:**
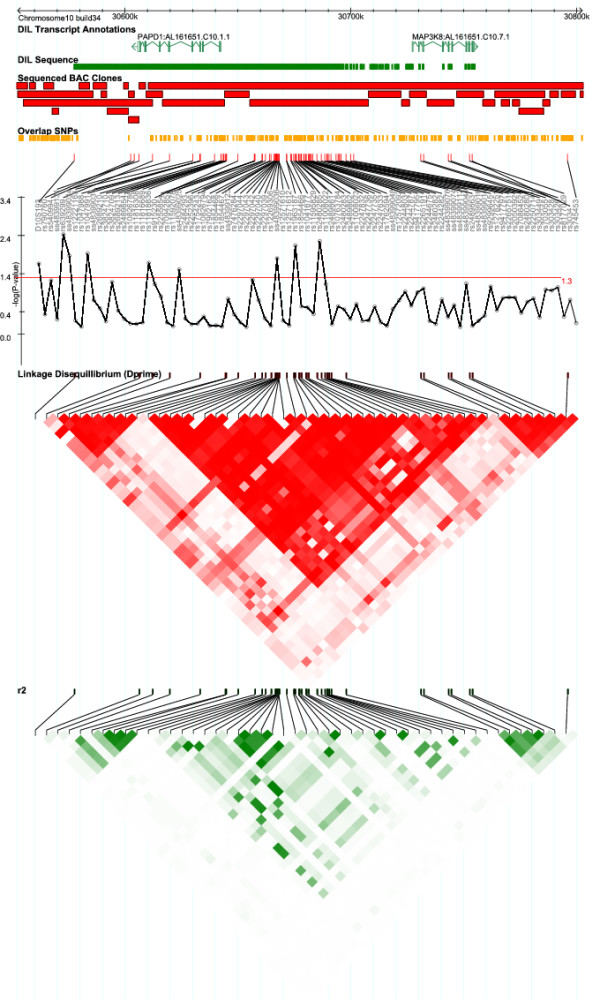
**The PAPD1-MAP3K8 gene region on chromosome 10**. Tracks indicate (from top to bottom): position in the human genome chromosome 10, NCBI build 34; annotated genes; regions where resequencing of the amplified fragments has been attempted; sequenced BAC clones; SNPs identified in the clone overlaps; polymorphisms genotyped in up to 765 UK and USA families with two affected offspring; SNP rs or ss numbers; a plot showing -log(*P*-value) for association in up to 765 families with red line at 1.3 corresponding to *P *= 0.05; linkage disequilibrium (LD) plots for SNPs with minor allele frequency > 0.1 showing pairwise LD by colour ranging from red (high D') or dark green (high r^2^), indicating strong LD, to white, indicating weak or no LD between SNPs. LD plots have been generated using Haploview [27].

Two genes localize within 150 kb of the D10S193, rs1963187 and rs2480285 markers (Figure 1 and [14]). The polyA polymerase associated domain containing 1 (PAPD1) gene encodes a protein with a nucleic acid binding PAP/25A-associated domain. Associated polymorphisms flank *PAPD1*, while the second gene, known as *MAP3K8 *(mitogen-activated protein kinase kinase kinase 8) is located 50 – 177 kb away from the associated polymorphisms. We then searched for novel sequence polymorphisms in this region. Using a panel comprising eight Caucasian individuals we resequenced 38.8 kb in the 177.2 kb region between D10S193 and exon 9 of the MAP3K8 gene. Additionally, to discover rarer genetic variants we resequenced exons, exon-intron boundaries and putative regulatory regions of the PAPD1 gene using a panel of 96 T1D patients (each representing one of the UK multiplex families in which affected sibs share both chromosomes identical-by-descent in the *IDDM10 *region). In total we found 147 SNPs (Supplementary Table 4, see Additional file 4).

Then, in addition to the five polymorphisms (two STRs and three SNPs) in the *PAPD1-MAP3K8 *region that were already genotyped, we tested 84 SNPs identified by resequencing. At first we studied association in 458 UK families (Supplementary Table 5, see Additional file 5). Subsequently, seven SNPs that were suggestively associated in these UK families (*P *= 0.012 – 0.073) were genotyped in the extended set of up to 2,857 T1D families. Thus, overall we studied 12 polymorphisms in the *PAPD1-MAP3K8 *region in all available T1D families. We found that alleles of six SNPs that localize in the PAPD1 gene show nominal evidence of association with T1D risk or protection (*P *= 0.0026 – 0.031, Table 1). Then we genotyped an additional sample of 1,693 unrelated T1D patients and 1,805 controls from the UK (Table 2) for six *PAPD1 *polymorphisms that were associated in the previous analysis of the extended family set. We found that only microsatellite marker D10S193 located 28 kb downstream of *PAPD1 *was weakly associated in this sample (*P *= 0.03, Table 2). Thus allele 226 of D10S193 was weakly associated with T1D risk both in the families (relative risk [RR] = 1.15, *P *= 0.019) and in the case-control analysis (OR = 1.16, *P *= 0.078). Another D10S193 allele 228 was associated with protection from T1D in cases and controls (OR = 0.73, *P *= 0.006), but not in the families (RR = 0.96, *P *= 0.59).

Additionally, we further studied seven SNPs in the MYO3A, HRNPF, NRP1 and SVIL gene regions that have shown some evidence of association in the T1D families (*P *= 0.0092 – 0.04, Table 1). We genotyped these SNPs in 1,693 T1D patients and 1,805 controls, but found no association with T1D (Table 2).

## Discussion

Overall, the association signals that we detected in the *IDDM10 *region near the PAPD1 gene did not reach genome-wide significance levels, despite having tested large samples of T1D families, cases and controls. This association could be spurious or could indicate a small genuine effect located near the PAPD1 gene that we did not have statistical power to demonstrate at a genome-wide significance level. Such an effect could not explain the reported evidence of T1D linkage at this region of chromosome 10, λs = 1.12. If *IDDM10 *is a true disease locus, it could be caused by a single common contributory variant with strong effect (such as OR = 2 and minor allele frequency of 0.2) or, more likely, by a number of variants with smaller effects located in this region. Therefore, further association studies in datasets that are powered to identify weak genetic effects (e.g. OR = 1.3 – 1.5) are needed to discover these type 1 diabetes genes.

## Conclusion

We identified a large number of SNPs for genetic studies in the *IDDM10 *region using a novel sequencing strategy, performed a first T1D association scan of this region and eliminated the possibility that two functional candidate genes, *CREM *and *SDF1*, have major effect on T1D. The weak association signal near the PAPD1 gene detected in the association scan may be either false or due to a small genuine effect, and cannot explain the previously observed strong linkage in the *IDDM10 *region.

## Methods

### Double tile path construction and shotgun sequencing

We constructed a double tile path between 21,022,316 and 44,323,745 bp of chromosome 10 (NCBI build 34) from two bacterial artificial chromosome (BAC) libraries, RPCI-11 and RPCI-13, and sequenced selected clones at the Wellcome Trust Sanger Institute. In total the shotgun tile paths comprised 110 RPCI-11 clones and 86 RPCI-13 clones that represent 12.6 Mb of overlap sequence. In addition to the finished sequence of chromosome 10, we shotgun sequenced to draft quality complementary clones from the other library in the tile path. In 1,060 overlaps the RPCI-11 clone was completely finished and in 76 overlaps the RPCI-13 clone was completely finished. Of the unfinished clones 77 (98.72%) were in multiple smaller contigs, i.e. draft quality.

### SNP identification

Repeats were masked using RepeatMasker [15] and then masked sequences were used in pair-wise sequence alignments by Sequence Search and Alignment by Hashing Algorithm (SSAHA) to map clone sequences and find SNPs [16]. Overlaps ≥ 2 kb were considered for SNP identification. We only checked overlaps between clones in different tile paths as overlaps from the finished tile path had been checked for SNPs previously. A file of overlap pairs was derived and used as an input for a script that calls SSAHA and then parses the resulting alignments. To avoid false SNP calling due to misalignment, clusters of five or more SNPs were rejected when each one was less then 10 bp away from neighboring SNPs. We then mapped SNPs on the human genome consensus path (NCBI build 34) using the mapping information from the clone and the SSAHA algorithm. The tile paths and SNPs can be viewed on our website [14]. Information on all polymorphisms has been submitted to dbSNP [17].

### Subjects

The study was done according to the principles of the Helsinki Declaration. We obtained permission from relevant ethical committees and informed consent from the participating subjects. Initially, we genotyped 329 polymorphisms in up to 765 families of Caucasian ethnic group, each with two children affected with T1D comprising 458 Diabetes UK Warren families and 307 The Human Biological Data Interchange (HBDI) families. An extended set of families with at least one affected child comprised families from the UK (n = 1,781, including 458 Diabetes UK Warren families), Norway (n = 359), Romania (n = 352) and the USA (n = 365, including 307 HBDI families). Therefore, in total we studied 2,857 T1D families; exact number of affected offspring genotyped for each SNP is shown in Table 1. Assuming multiplicative genetic model, this extended set of T1D families provides over 80% power to detect genetic effect with odds ratios (OR) of 1.25 and 1.5 for alleles at 40% and 7%, respectively, at  α = 10^-6^. Additionally we studied an independent sample of 1,693 T1D patients collected across the UK and 1,828 control subjects that were selected from the 1958 British Birth cohort [18]. This case-control collection would have over 99% power to detect OR = 1.25 and 1.5 for alleles at 40% and 7%, respectively, at α = 0.05.

### Candidate gene resequencing and genotyping

We designed primers using Primer3 [19], amplified genomic DNA by PCR and sequenced PCR fragments with an ABI Big Dye Terminator v3.1 kit and an ABI3700 capillary sequencer (ABI, Foster City, CA). Sequence reads were aligned using the Staden package [20]. We resequenced in 32 individuals all *CREM *and *SDF1 *exons, exon-intron boundaries, and up to 3 kb upstream and downstream of the gene. All identified SNPs have been submitted to dbSNP [17]. SNP genotyping was carried out using Invader (Third Wave Technologies, Madison, WI), TaqMan (Perkin Elmer Applied Biosystems, Foster City, CA) or BeadArray (Illumina Inc, San Diego, CA). Microsatellite markers were genotyped as described elsewhere [13].

### Statistical analysis

We assessed genotype frequency among parents for each polymorphism using Arlequin version 2.000 [21] and found no unexpected deviation from the Hardy-Weinberg equilibrium (*P *> 0.01). Statistical analysis was carried out within STATA version 8.1 [22]. Tag SNPs that capture common allelic variation (MAF > 0.03) with r^2 ^≥ 0.8 were selected using htstep, htsearch and haptag programs within Stata [23,24]. When a tag SNP approach was taken, we used a global association multilocus test using mlpop program in Stata. It tests for association between the disease and the tag SNPs due to linkage disequilibrium with one or more causal variants in the region. This test contrasts the allele frequencies of a non-redundant set of tag SNPs between cases and controls by use of Hotelling's *T*^2 ^test [25,26]. We did not apply multiple testing corrections in this study and all *P*-values reported are uncorrected.

## Abbreviations

BAC – bacterial artificial chromosome, HBDI – Human Biological Data Interchange, MAF – minor allele frequency, MAP3K8 – mitogen-activated protein kinase kinase kinase 8, OR – odds ratio, PAPD1 gene – polyA polymerase associated domain containing 1 gene, RR – relative risk, SDF1 – stromal cell-derived factor 1, SNP – single nucleotide polymorphism, SSAHA – Sequence Search and Alignment by Hashing Algorithm, STRs – short tandem repeats, T1D – type 1 diabetes.

## Authors' contributions

S Nejentsev contributed to the design of the study, genotyping, sequencing, data analysis and drafted the manuscript. LJS participated in SNP identification, bioinformatics analysis and drafting of the manuscript. DS, RB, CEL, FP, JM and LG contributed to genotyping. NMW was responsible for the data management. AL and OB contributed to bioinformatics analysis. HS and S Nutland were responsible for the DNA. AS, RT and BJB contributed to the study design. CW participated in contig assembly, genomic sequencing and analysis and SNP genotyping. LF participated in contig assembly and genomic sequencing. YC identified SNPs from initial genomic sequencing. PD and JR supevised contig assembly and genomic sequencing. ID participated in the design of the study, oversight of the contig assembly and genomic sequencing, obtaining funding for the study, data analysis, and manuscript preparation. JAT participated in the design of the study, obtaining funding for the study, data analysis, and manuscript preparation. All authors read and approved the final manuscript.

## Supplementary Material

Additional File 1 Supplementary Table 1 Click here for file

Additional File 2 Supplementary Table 2 Click here for file

Additional File 3 Supplementary Table 3 Click here for file

Additional File 4 Supplementary Table 4 Click here for file

Additional File 5 Supplementary Table 5 Click here for file
